# Baseline OCT Biomarkers and Early Outcomes After Idiopathic Full-Thickness Macular Hole Surgery: Associations with Primary Failure and Visual Acuity After Successful Closure

**DOI:** 10.3390/diagnostics16203319

**Published:** 2026-10-13

**Authors:** Luca Timoceanu, Corina Lang, Scott Tschuppert, Josef Guber, Christophe Valmaggia, Andreas Ebneter

**Affiliations:** 1 Department of Ophthalmology, Cantonal Hospital St. Gallen, 9007 St. Gallen, Switzerland; 2 Faculty of Medicine, University of Bern, 3008 Bern, Switzerland; 3 Department of Ophthalmology, Cantonal Hospital St. Gallen, University of Bern, 3008 Bern, Switzerland

**Keywords:** idiopathic full-thickness macular hole, optical coherence tomography, vitrectomy, visual outcomes, biomarkers, prognosis

## Abstract

**Background/Objectives**: Baseline visual acuity (VA) and minimum linear diameter (MLD) are well-established predictors after surgery for idiopathic full-thickness macular hole (FTMH). It is less clear whether other optical coherence tomography (OCT) features provide additional information. We examined the associations of selected baseline OCT biomarkers with primary anatomical failure and with early postoperative VA after successful closure. **Methods**: We retrospectively studied eyes that underwent surgery for idiopathic FTMH at a single Swiss center between 2019 and 2024. Primary failure was assessed exploratorily using Firth penalized logistic regression. Early postoperative VA was analyzed among eyes with successful primary closure and an eligible visit 21–60 days after surgery. Linear regression models included baseline VA and MLD, with each OCT biomarker added separately. Patient-level cluster-robust standard errors accounted for bilateral inclusion. **Results**: This study included 127 eyes from 124 patients. Primary closure was achieved in 116 eyes (91.3%), while 11 eyes (8.7%) had primary failure. Greater MLD was associated with higher odds of primary failure (odds ratio per 100 µm, 2.09; 95% confidence interval, 1.46–3.26). Among the 99 successfully closed eyes with eligible early follow-up, several OCT biomarkers were associated with postoperative VA in univariable analyses. However, none remained significant after adjustment for baseline VA and MLD and correction for multiple testing. Bumpy borders produced the largest, although modest, improvement in model performance. **Conclusions**: MLD showed the most consistent exploratory association with primary failure. Additional OCT biomarkers provided limited independent information about early postoperative VA beyond baseline VA and MLD in eyes with successful primary closure and eligible early follow-up.

## 1. Introduction

Full-thickness macular hole (FTMH) is a foveal disorder that typically causes reduced central visual acuity (VA) and metamorphopsia [1]. Since the introduction of modern vitreoretinal surgery, the outlook for affected eyes has improved markedly. Pars plana vitrectomy (PPV) combined with internal limiting membrane (ILM) peeling and gas tamponade now achieves high closure rates, and most patients experience visual improvement after surgery [2,3]. Even so, postoperative recovery is not uniform. Some eyes recover quickly and gain substantial vision, whereas others improve only modestly despite successful closure. This variability matters in everyday practice, where patients want realistic counseling before surgery.

The best established preoperative predictors of outcome are baseline VA and macular hole size, particularly minimum linear diameter (MLD) [4]. These parameters are useful, but they do not capture the full structural spectrum of idiopathic FTMH. Spectral-domain optical coherence tomography (OCT) offers a more detailed view of foveal morphology and has therefore drawn attention to additional features that may reflect chronicity, tractional configuration, or tissue damage [5]. Proposed biomarkers include intraretinal cystic changes [6], vitreomacular traction (VMT) [7], bumpy borders, supra-retinal pigment epithelium (RPE) granular deposits [8], epiretinal proliferation (ERP) [9] and disruption of the outer retinal layers [10]. Several of these findings have been linked to postoperative outcomes, but the literature is mixed, and it remains unclear whether they show independent associations with postoperative visual function once baseline VA and MLD are already known [11]. Rather than evaluating these OCT features as isolated predictors, the present study assessed them within a common analytical framework and examined whether they added information beyond baseline VA and MLD. Selected previous studies evaluating the prognostic value of additional OCT biomarkers and their reported effect estimates are summarized in Supplementary Table S1.

Another issue is that anatomical and functional outcomes should not automatically be treated as the same question. Some baseline features may be more relevant to hole closure itself, whereas others may matter more for visual recovery after closure has been achieved [12,13]. A single postoperative VA model that pools all operated eyes can blur this distinction. Clinically, however, these are separate questions: which eyes are at higher risk of primary anatomical failure, and among eyes that do close, which factors are associated with postoperative visual function? An analysis restricted to successfully closed eyes answers the second question conditionally and should not be interpreted as estimating visual outcome for the entire surgical cohort.

We therefore examined the associations of baseline OCT biomarkers with two distinct early outcomes after surgery for idiopathic FTMH. Primary failure was analyzed in the full included cohort as an exploratory anatomical outcome. Early postoperative VA was analyzed conditionally among eyes that achieved primary closure and had an eligible assessment between 21 and 60 days after surgery. This separation allowed anatomical failure and visual recovery after successful closure to be addressed as distinct clinical questions. This early interval was selected because it represented the most consistently available postoperative assessment in the cohort and permitted analysis of a relatively homogeneous follow-up period. We specifically assessed whether selected preoperative OCT features remained independently associated with early postoperative VA after accounting for baseline VA and MLD.

## 2. Materials and Methods

### 2.1. Study Design and Setting

This retrospective observational cohort study included eyes that underwent surgery for idiopathic FTMH at the Cantonal Hospital of St. Gallen, Switzerland, between January 1, 2019 and December 31, 2024. This study adhered to the Declaration of Helsinki and was approved by the local ethics committee (Ethikkommission Ostschweiz; Ref. 2025-02161). Written informed consent for the use of clinical data was obtained in accordance with local regulations.

### 2.2. Study Population

Consecutive eyes were eligible if baseline clinical data, preoperative OCT imaging and at least 1 postoperative assessment performed 21 days or later after surgery were available. Both eyes of the same patient could be included if each eye fulfilled the eligibility criteria. Eyes with secondary FTMH related to rhegmatogenous retinal detachment, high myopia (axial length > 26.5 mm or spherical equivalent ≤ −6.00 diopters), ocular trauma, prior PPV, or other vision-impairing ocular disease apart from FTMH or cataract were excluded.

For the early functional analysis, eyes also required successful primary closure and at least 1 eligible postoperative visit between 21 and 60 days after surgery. Accordingly, this analysis was conditional on both primary closure and availability of an eligible early follow-up assessment. Successfully closed eyes without an eligible visual-acuity assessment between postoperative days 21 and 60 were excluded from the functional regression analysis but remained part of the anatomical cohort and descriptive postoperative analyses where applicable.

### 2.3. Clinical and Visual Acuity Data

Baseline variables included age, sex, laterality, lens status, symptom duration and preoperative VA. Symptom duration was based on the patient-reported time from onset of visual symptoms to FTMH diagnosis, as recorded in the earliest available clinical note or referral letter. Monocular distance VA was assessed using a Snellen decimal chart under routine standardized examination conditions. Pinhole testing or manifest refraction was performed when required to obtain the best-recorded distance VA. Values were converted to logMAR for analysis. For low-vision categories, counting fingers, hand motions, light perception and no light perception were converted to logMAR values of 2.1, 2.4, 2.7 and 3.0, respectively [14].

### 2.4. OCT Imaging and Biomarker Assessment

**Figure 1:**
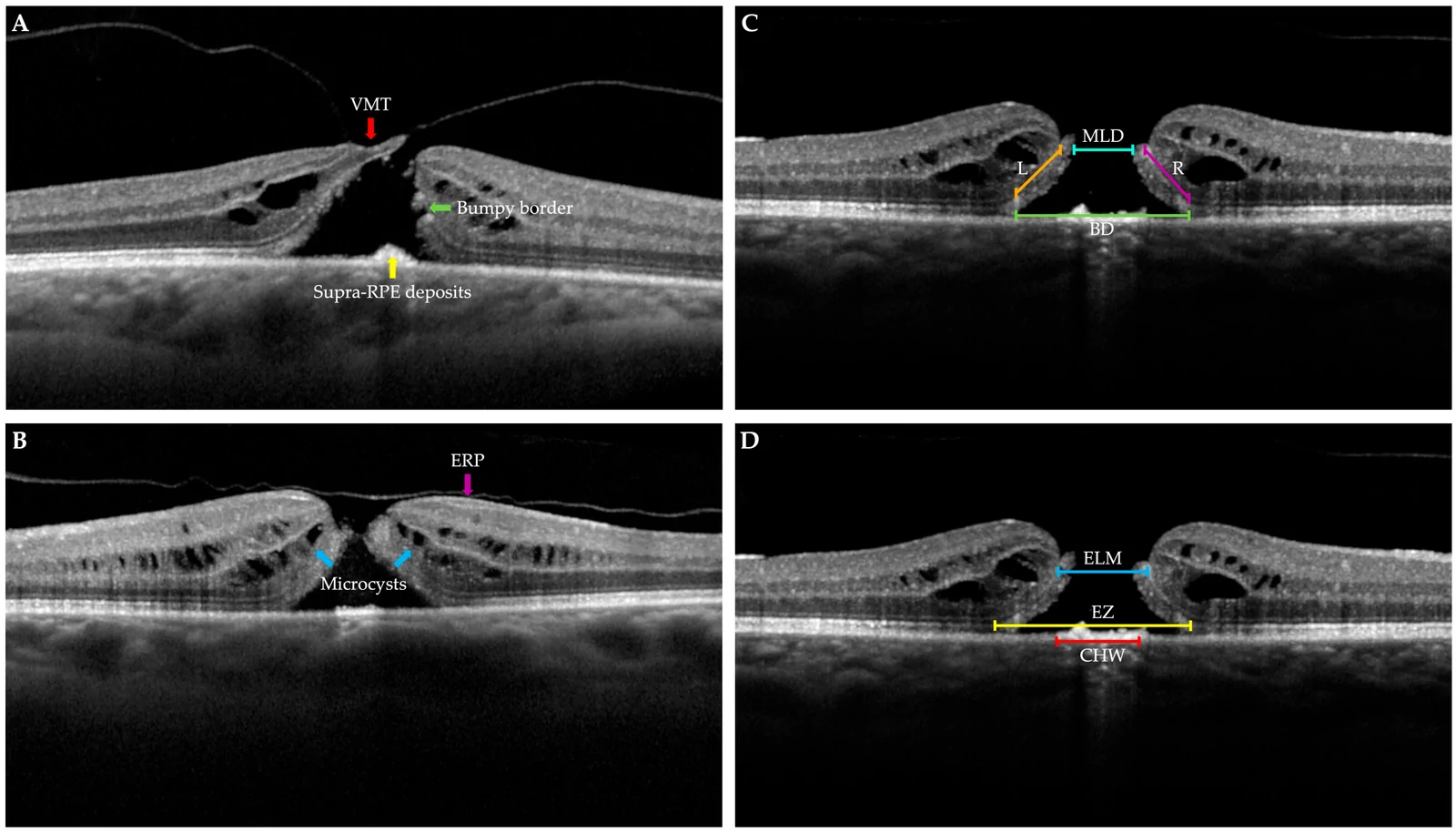
Representative optical coherence tomography features and measurement methods evaluated in this study. (**A**) Representative preoperative OCT image showing vitreomacular traction (VMT, red arrow), bumpy border (green arrow), and supra-RPE granular deposits (yellow arrow). (**B**) Representative preoperative OCT image showing microcysts (blue arrows) and epiretinal proliferation (ERP, purple arrow). (**C**) Representative preoperative OCT image showing basal diameter (BD, green line), minimum linear diameter (MLD, cyan line), and the detached retinal lengths on the left (L, orange line) and right (R, purple line) used to calculate the foveal actual defect (FAD). FAD was calculated as BD − (L + R). (**D**) Representative preoperative OCT image showing external limiting membrane (ELM) disruption (blue line), ellipsoid zone (EZ) disruption (yellow line), and choroidal hypertransmission width (CHW, red line).

The recorded OCT features included VMT, microcysts, bumpy borders, supra-RPE granular deposits, ERP, MLD [15], basal diameter (BD), foveal actual defect (FAD) [16], external limiting membrane (ELM) disruption, ellipsoid zone (EZ) disruption and choroidal hypertransmission width (CHW). VMT was defined as persistent focal attachment of the posterior hyaloid with associated distortion or elevation of the foveal tissue. Microcysts were defined as well-demarcated hyporeflective intraretinal cystic spaces adjacent to the macular hole. Bumpy borders were defined as irregular or undulating hole margins with visible projections, lobulations or indentations. Supra-RPE granular deposits were defined as discrete hyperreflective granular foci immediately above the RPE at the base of the hole. ERP was defined as homogeneous material of intermediate reflectivity on the inner retinal surface, contiguous with the hole margin and distinguishable from a conventional tractional epiretinal membrane. MLD was measured as the narrowest full-thickness distance between the opposing retinal edges, parallel to the RPE. BD was measured between the nasal and temporal points where retinal detachment from the RPE began. FAD was calculated as the basal diameter minus the sum of the detached nasal and temporal retinal lengths: FAD = BD − (nasal detached retinal length + temporal detached retinal length), as previously described [16]. ELM and EZ disruption were measured as the horizontal distance between the nearest identifiable ends of the respective retinal layers. CHW was measured as the horizontal width of increased OCT signal transmission into the choroid beneath the macular hole. Representative examples and measurement methods are shown in Figure 1.

All OCT examinations were acquired using spectral-domain OCT (Spectralis; Heidelberg Engineering, Heidelberg, Germany) on the HEYEX 2 platform, version 2.5.4. Imaging comprised 6 to 24 radial B-scans centered on the fovea, with additional high-density macular cube scans obtained when clinically required. Images were assessed for adequate signal, foveal centration and major artifacts. Eyes without baseline scans of sufficient quality for reliable grading were excluded during cohort selection. OCT biomarkers were graded by a single grader (LT) masked to postoperative outcomes. Operational definitions and assessment methods for the evaluated OCT biomarkers are provided in Supplementary Table S2.

### 2.5. Surgical Procedure

All eyes underwent 23-gauge PPV under general anesthesia using a non-contact wide-angle viewing system (Oculus BIOM, Oculus Optikgeräte GmbH, Wetzlar, Germany or Zeiss RESIGHT, Carl Zeiss Meditec AG, Jena, Germany). Core vitrectomy was followed by induction of posterior vitreous detachment when absent. The ILM was stained with a dual dye containing DDG and trypan blue. Conventional ILM peeling over approximately 2 to 3 disc diameters around the fovea was performed in most eyes, while a free ILM flap technique was used in five primary procedures at the discretion of the operating surgeon. After fluid–air exchange, 20% sulfur hexafluoride (SF6) or 12% perfluoropropane (C3F8) was used at the surgeon’s discretion. SF6 was the routine tamponade, whereas C3F8 was reserved for selected challenging cases. Face-down positioning was prescribed for 0 to 8 days according to surgeon judgment, and adherence was not objectively monitored. All phakic eyes underwent combined phacoemulsification and intraocular lens implantation. Reoperations for primary failure were performed using either a free ILM flap technique or repeat PPV without a free flap, according to the intraoperative findings and surgeon judgment.

### 2.6. Outcomes and Follow-Up

Postoperative data were available for up to three follow-up visits and included follow-up time, postoperative VA, ELM disruption, EZ disruption and CHW.

The anatomical outcome was primary closure status after the initial surgery, determined at the first postoperative OCT assessment at which closure status was documented after the primary procedure. Primary closure was defined as restoration of the foveal contour with complete approximation of the neurosensory retina. Eyes not meeting this definition were classified as primary failure. Eyes that achieved anatomical closure only after reoperation remained classified as primary failures for the primary anatomical analysis. The main functional outcome was early postoperative VA in eyes with successful primary closure. This analysis was restricted to eyes with at least 1 eligible postoperative visit between 21 and 60 days after surgery. When more than 1 eligible visit was available within this window, the visit closest to postoperative day 30 was selected. The functional endpoint was VA (logMAR) at this selected visit. The functional analysis was therefore performed in the subset of eyes with successful primary closure and an eligible early postoperative visit. It did not estimate postoperative visual outcomes among eyes with primary failure or among successfully closed eyes without an eligible visit in the prespecified window.

### 2.7. Statistical Analysis

Descriptive statistics were used to summarize baseline, perioperative and postoperative characteristics. Baseline characteristics were reported for the full cohort and separately by closure status. Categorical variables were summarized as number and percentage, and continuous variables as mean ± standard deviation (SD) or median and interquartile range (IQR), as appropriate.

To assess potential selection associated with early follow-up availability, baseline and perioperative characteristics were compared between successfully closed eyes included in the 21- to 60-day functional analysis and successfully closed eyes without an eligible assessment in this interval. Continuous and categorical differences were summarized using absolute standardized mean differences. These comparisons were descriptive and were not subjected to formal hypothesis testing.

To examine associations with primary failure, exploratory Firth penalized logistic regression models were fitted with primary failure coded as 1 and primary closure as 0. Because only 11 eyes had primary failure, model complexity was kept deliberately limited to reduce the risk of overfitting. The primary adjusted model included MLD as the core covariate, and biomarker-specific models were fitted by adding 1 baseline OCT biomarker at a time. Results are reported as odds ratios (ORs) with 95% confidence intervals (CIs). Penalized likelihood-ratio tests were used for biomarker-specific inference, and Holm adjustment was applied separately within each family of exploratory biomarker analyses.

Associations with early postoperative VA were examined conditionally in eyes with successful primary closure and a selected postoperative visit between 21 and 60 days after surgery. Univariable linear regression models were fitted, followed by core-plus-one-biomarker models adjusted for baseline VA and MLD. Regression coefficients with 95% CIs were estimated using patient-level cluster-robust standard errors. Incremental model value was summarized using changes in adjusted R^2^, Akaike information criterion and root mean square error, with core and extended models compared on identical samples. Correlations and variance inflation factors were examined to assess overlap among baseline VA, MLD and continuous OCT measures. Sensitivity models additionally adjusted for age, baseline phakic status, tamponade type and exact postoperative assessment day. Exploratory associations between symptom duration and baseline OCT biomarkers were assessed using Spearman correlation or Mann–Whitney U tests, as appropriate, with Holm correction for multiple testing. Functional models were additionally repeated in eyes with documented symptom duration, including log2-transformed symptom duration as a covariate. However, these analyses could not address possible selection related to missing early follow-up, as postoperative VA was not available for the excluded eyes.

The primary functional models included 99 eyes from 97 patients with complete data for the outcome, predictors and sensitivity covariates. The symptom-duration sensitivity analysis included 46 eyes with documented symptom duration. Statistical analyses were performed in Python 3.9.2 using pandas, scipy, statsmodels and firthlogist. All tests were 2-sided and *p* < 0.05 was considered statistically significant.

## 3. Results

### 3.1. Study Cohort and Baseline Characteristics

A total of 127 eyes from 124 patients were included. Bilateral inclusion occurred in three patients (2.4%). Mean age at diagnosis was 69.6 ± 7.6 years, mean baseline VA was 0.79 ± 0.40 logMAR, and MLD was 305.1 ± 175.8 µm. Based on MLD, 54 eyes (42.5%) were classified as small (≤250 µm), 39 (30.7%) as medium (>250 to ≤400 µm) and 34 (26.8%) as large (>400 µm). Baseline clinical and OCT characteristics are summarized in Table 1.

### 3.2. Perioperative Characteristics

All 91 phakic eyes (71.7%) underwent combined phacoemulsification with intraocular lens implantation. A free ILM flap technique was used during the primary operation in five eyes (3.9%), all of which achieved primary closure. Intraocular tamponade consisted of SF6 in 116 eyes (91.3%) and C3F8 in 11 eyes (8.7%). Primary closure occurred in 108 of 116 SF6-treated eyes and eight of 11 C3F8-treated eyes. Therefore, tamponade type was not perfectly collinear with primary closure status. Mean face-down positioning duration was 4.7 ± 2.2 days.

### 3.3. Anatomical Outcome

Primary closure was achieved in 116 of 127 eyes (91.3%), whereas 11 eyes (8.7%) were classified as primary failure. Of the 11 eyes with primary failure, seven (63.6%) underwent reoperation. Six were treated using a free ILM flap technique, and one underwent repeat PPV without a free flap. All seven reoperated eyes subsequently achieved anatomical closure. The remaining four eyes (36.4%) did not undergo reoperation. Eyes that closed following reoperation remained classified as primary failures because closure had not been achieved after the initial procedure. Of the 116 successfully closed eyes, 99 had an eligible postoperative assessment between 21 and 60 days and were included in the conditional functional analysis. The remaining 17 successfully closed eyes did not have an eligible visit within this interval and were excluded from the functional regression analysis. Baseline characteristics stratified by closure status are presented in Supplementary Table S3. Compared with eyes that achieved primary closure, eyes with primary failure tended to be older and to have a less favorable baseline profile, including worse baseline VA, larger hole dimensions, more outer retinal disruption and greater CHW. Large holes (>400 µm) were also more frequent in eyes with primary failure.

### 3.4. Comparison of Successfully Closed Eyes Included and Excluded from the Functional Analysis

Baseline and perioperative characteristics of the 99 included and 17 excluded successfully closed eyes are presented in Supplementary Table S4. Several observed differences were present between the groups. Compared with excluded eyes, included eyes were older (69.5 versus 66.4 years; absolute standardized mean difference [SMD], 0.45), had worse preoperative VA (0.79 versus 0.61 logMAR; SMD, 0.52), and had larger MLD (295.2 versus 217.9 µm; SMD, 0.49). Included eyes also had larger FAD and more extensive ELM disruption and choroidal hypertransmission, whereas VMT was less frequent (29.3% versus 58.8%). These differences indicate that availability of an eligible early postoperative assessment may have selected a clinically and morphologically different subgroup of successfully closed eyes.

### 3.5. Exploratory Associations with Primary Failure

Because primary failure occurred in only 11 eyes, analyses for this endpoint were treated as exploratory and performed using Firth penalized logistic regression (Table 2). In the core model, greater MLD was associated with higher odds of primary failure (OR per 100 µm, 2.09; 95% CI, 1.46–3.26; *p* < 0.001). In separate core-plus-one-biomarker models adjusted for MLD, microcysts showed a nominal association with lower odds of primary failure (OR, 0.09; 95% CI, 0.01–0.71; *p* = 0.024), whereas bumpy borders (OR, 9.51; 95% CI, 1.05–1255.25; *p* = 0.043) and preoperative CHW (OR per 100 µm, 2.02; 95% CI, 1.02–4.31; *p* = 0.045) showed nominal associations with higher odds of primary failure. The estimate for bumpy borders was highly imprecise, as reflected by very wide CIs. No significant adjusted associations were observed for VMT, supra-RPE granular deposits, ERP, BD, FAD, preoperative ELM disruption, or preoperative EZ disruption. None of the biomarker associations remained significant after Holm correction for multiple testing.

### 3.6. Postoperative Assessment

Postoperative outcomes by follow-up interval are shown in Table 3 for all eyes and in Supplementary Table S5 for eyes with successful primary closure. A total of 107 eyes had an eligible visual-acuity assessment between postoperative days 21 and 60. Of these, 99 had achieved primary closure and were included in the conditional functional analysis, whereas eight had primary failure and contributed only to the descriptive postoperative summary. In the full cohort, mean postoperative VA was 0.47 ± 0.34 logMAR at 21 to 60 days, 0.31 ± 0.26 logMAR at 61 to 180 days, 0.18 ± 0.12 logMAR at 181 to 365 days and 0.35 ± 0.58 logMAR beyond 365 days. In eyes with successful primary closure, corresponding mean postoperative VA values were 0.43 ± 0.31, 0.20 ± 0.15, 0.18 ± 0.12 and 0.17 ± 0.14 logMAR, respectively. Most successfully closed eyes contributed to the 21- to 60-day interval (99 eyes), whereas substantially fewer observations were available at later time points. Later follow-up summaries should therefore be interpreted descriptively.

### 3.7. Associations with Early Postoperative Visual Acuity Among Successfully Closed Eyes

Linear regression analyses of early postoperative VA at the selected 21- to 60-day follow-up visit in eyes with successful primary closure are summarized in Table 4. A total of 99 eyes were analyzed. These results are conditional on successful primary closure and the availability of an eligible early postoperative visit and should not be interpreted as visual outcomes for the full surgical cohort. In the prespecified core model, neither baseline VA nor MLD was independently associated with early postoperative VA (baseline VA per 0.1 logMAR worse: β = 0.0214; 95% CI, −0.0017 to 0.0446; *p* = 0.069; MLD per 100 µm: β = 0.0465; 95% CI, −0.0313 to 0.1243; *p* = 0.238). Baseline VA and MLD were moderately correlated (Pearson r = 0.66; Spearman ρ = 0.61; both *p* < 0.001), although variance inflation factors were low at 1.77 for each variable. Several quantitative OCT measures were more strongly intercorrelated, particularly MLD, FAD, ELM disruption and CHW. Detailed correlation, variance-inflation, model-performance and sensitivity results are provided in Supplementary Tables S6A–S6D.

In univariable analyses, worse early postoperative VA was associated with worse baseline VA (β per 0.1 logMAR, 0.0349; 95% CI, 0.0214 to 0.0484; *p* < 0.001), greater MLD (β per 100 µm, 0.0788; 95% CI, 0.0285 to 0.1292; *p* = 0.002), the presence of microcysts (β = 0.1578; 95% CI, 0.0539 to 0.2616; *p* = 0.003), bumpy borders (β = 0.1797; 95% CI, 0.0665 to 0.2929; *p* = 0.002), larger BD (β per 100 µm, 0.0404; 95% CI, 0.0095 to 0.0712; *p* = 0.011), larger FAD (β per 100 µm, 0.0738; 95% CI, 0.0302 to 0.1174; *p* = 0.001), greater preoperative ELM disruption (β per 100 µm, 0.0659; 95% CI, 0.0247 to 0.1070; *p* = 0.002), greater preoperative EZ disruption (β per 100 µm, 0.0255; 95% CI, 0.0081 to 0.0429; *p* = 0.005) and greater preoperative CHW (β per 100 µm, 0.0653; 95% CI, 0.0127 to 0.1179; *p* = 0.015), whereas ERP was associated with better early postoperative VA (β = −0.1481; 95% CI, −0.2592 to −0.0371; *p* = 0.009). After Holm correction, the univariable associations for microcysts, bumpy borders, BD, FAD, preoperative ELM disruption, preoperative EZ disruption, preoperative CHW and ERP remained significant.

In core-plus-one-biomarker models, no OCT biomarker remained significantly associated with early postoperative VA after Holm correction. Bumpy borders showed the strongest nominal association with worse VA (β = 0.1076; 95% CI, 0.0100 to 0.2053; *p* = 0.031; Holm-adjusted *p* = 0.311) and produced the largest, although modest, improvement in model performance (change in adjusted R^2^, +0.021; change in AIC, −1.65). ERP produced a smaller improvement, whereas the remaining biomarkers added little model information. Results were materially unchanged in sensitivity models additionally adjusted for age, phakic status, tamponade type and the timing of the postoperative assessment. In separate exploratory analyses, longer symptom duration was associated with greater BD, FAD, ELM disruption and EZ disruption after Holm correction (Supplementary Table S7). A further analysis restricted to 46 eyes with available symptom-duration data showed similar functional findings. In the functional sensitivity analyses, no biomarker remained significant after Holm correction.

## 4. Discussion

This study included 127 eyes from 124 patients undergoing surgery for idiopathic FTMH, with primary closure achieved in 116 eyes (91.3%). Several baseline OCT biomarkers were associated with early postoperative VA in univariable analyses, but none remained statistically significant after adjustment for baseline VA and MLD and correction for multiple testing. This finding applies only to eyes that achieved primary closure and had an eligible assessment between 21 and 60 days after surgery. In the separate exploratory anatomical analysis, MLD showed the most consistent association with primary failure among the limited models fitted.

This overall pattern fits previous work on macular hole surgery. Modern PPV with ILM peeling and gas tamponade generally produces high anatomical success rates, and hole size remains one of the strongest preoperative determinants of closure [12,17]. In our cohort, eyes with primary failure also tended to have a less favorable baseline profile, including worse VA, larger MLD, larger BD, larger FAD, more pronounced outer retinal alteration and greater CHW. Taken together, this suggests that eyes with primary failure entered surgery with more advanced structural disease. Across the limited core-plus-one-biomarker models, MLD showed the most consistent association with primary failure, in keeping with earlier studies and meta-analytic data identifying hole size as an important anatomical predictor [18,19].

The functional analysis showed a related, but not identical, pattern. In univariable models, several biomarkers were linked to worse early postoperative VA, including microcysts, bumpy borders, larger FAD and greater preoperative ELM and EZ disruption. ERP pointed in the opposite direction. Once baseline VA and MLD were added, these associations weakened and none remained significant after correction for multiple testing. Baseline VA and MLD were moderately correlated but did not show problematic variance inflation, whereas several quantitative OCT measures were highly correlated with MLD and with one another. This suggests that several OCT biomarkers may capture the same underlying structural severity as MLD rather than provide fully independent information. Once baseline VA and MLD were included, part of the association seen in the univariable models was therefore accounted for by these established predictors. The modest size of the functional cohort may also have reduced the precision of the adjusted estimates. Incremental model-performance gains were also generally small, although bumpy borders and ERP showed modest improvements. The absence of statistical significance should therefore not be interpreted as definitive evidence that the biomarkers have no independent or incremental value. That interpretation is also consistent with the broader literature, where associations between preoperative OCT features and postoperative VA have been reported, but their incremental value beyond established core predictors has varied from study to study [13,20,21].

Intraretinal cystic change has been linked to closure behavior and postoperative photoreceptor damage [22,23]. ERP may represent a distinct remodeling response [24]. Outer retinal integrity, particularly at the level of the ELM and EZ, has repeatedly been associated with visual prognosis after macular hole repair [25,26]. Quantitative OCT studies have likewise suggested that related structural indices may help explain postoperative visual recovery [27,28]. In the present study, bumpy borders showed the strongest nominal adjusted association, while ERP showed a favorable univariable signal, but neither remained significant after Holm correction. These findings were therefore insufficient to support their routine use as standalone markers for preoperative counseling [13,20,28].

The choice of endpoint is important when comparing our results with earlier reports. Many previous studies examined later or final postoperative VA, whereas our analysis focused deliberately on early postoperative VA at a selected visit between 21 and 60 days after surgery [29,30]. This distinction likely matters. Early visual recovery is influenced not only by preoperative morphology but also by short-term postoperative factors and by the pace of structural restoration after closure. Residual intraocular gas, recovery after combined phacoemulsification, transient postoperative retinal changes and variation in the timing of assessment may also influence VA during this interval. In contrast, later visual recovery may depend more on continued restoration of the outer retina, including the ELM and EZ. However, additional adjustment for age, phakic status, tamponade type and assessment day did not materially change the biomarker estimates. Biomarkers related to photoreceptor recovery or outer retinal repair may therefore show stronger associations with later visual outcomes than with the early endpoint used here [26,31]. Our findings should therefore not be assumed to apply to longer-term visual outcomes.

A strength of this study is that anatomical and functional outcomes were analyzed separately. However, the functional analysis was conditional on both successful primary closure and availability of an eligible early assessment. The 17 excluded closed eyes differed from the 99 included eyes in several baseline characteristics, and baseline OCT features may themselves influence primary closure. The functional cohort was therefore not fully representative of all operated or successfully closed eyes, and the findings should be interpreted as conditional associations rather than estimates of visual prognosis for the entire surgical cohort. Selection related to early follow-up availability may therefore have influenced the observed associations and may limit the generalizability of the functional analysis. This cannot be fully addressed by adjustment within the included cohort.

Because later follow-up was sparse, the functional models address early postoperative recovery only and do not exclude associations with longer-term recovery, particularly for biomarkers of outer retinal integrity [25,31].

Clinically, the message is necessarily cautious. For anatomical counseling before surgery, MLD remains an established OCT-derived metric and showed the most consistent exploratory association with primary failure in this cohort, in keeping with current practice and published evidence [18,19]. Additional OCT biomarkers may still help describe structural severity, but this study found limited evidence that they were independently associated with early VA among successfully closed eyes after accounting for baseline VA and hole size. Whether selected biomarkers can add meaningful clinical information will need to be tested in larger datasets with standardized follow-up and adequately powered multivariable analyses. Given the low primary failure rate, future validation will likely require multicenter cohorts and standardized assessment of both anatomical and longer-term visual outcomes.

### Limitations

Several limitations should be considered. First, the retrospective design carries a risk of selection bias and incomplete data capture. Symptom duration was unavailable for approximately half of the cohort and, as it was based on patient-reported symptom onset rather than documented hole onset, should be considered an approximate measure of FTMH chronicity. Second, only 11 eyes had primary failure, so the anatomical regression models should be interpreted as exploratory. Third, follow-up was not standardized, and relatively few eyes contributed data at later time points, limiting assessment of longer-term functional prognosis. The functional analysis was conditional on successful primary closure and availability of a 21- to 60-day postoperative assessment and therefore does not estimate visual outcomes for the entire surgical cohort. Seventeen successfully closed eyes lacked an eligible assessment within this interval. These eyes differed from included eyes in several measured baseline characteristics, including age, baseline VA, VMT, hole dimensions and outer retinal structural measures, suggesting that follow-up availability was not entirely random. Selection related to both primary closure and early follow-up availability may therefore have altered the associations observed in the functional models. Moreover, because OCT characteristics may influence primary closure, conditioning on closure itself may have altered the associations observed in the functional models. Fourth, several quantitative OCT measures were highly intercorrelated, particularly MLD, FAD, ELM disruption and CHW, limiting their statistical separation in a modest sample. Fifth, surgical management was not fully standardized. Tamponade type and the duration of face-down positioning were chosen by the operating surgeon, and a free ILM flap was used in a small number of primary procedures. These decisions may have been influenced by hole size or perceived surgical difficulty and may also have affected anatomical closure or early visual recovery. Although the sensitivity analyses adjusting for phakic status, tamponade type and assessment timing produced similar functional results, residual confounding cannot be excluded. With only 11 primary failures, the anatomical models could not support broader adjustment for surgical factors. The crude differences between tamponade groups should therefore not be interpreted causally. Sixth, OCT grading was performed by a single grader, and intra- and inter-grader reproducibility were not assessed. This is particularly relevant for qualitative features such as bumpy borders, supra-RPE granular deposits and ERP, for which grading may be more subjective. Future studies could strengthen reproducibility by using masked multi-grader assessment with agreement testing. Automated or semi-automated OCT measurements may also reduce grader-dependent variability, particularly for quantitative biomarkers. Finally, this study reflects the experience of a single center and may not be fully generalizable to other settings.

## 5. Conclusions

In summary, several baseline OCT biomarkers were associated with early postoperative VA in univariable analyses. However, these associations weakened after accounting for baseline VA and MLD and correcting for multiple testing in eyes with successful primary closure and eligible early follow-up. MLD showed the most consistent exploratory association with primary failure, although inference was limited by the small number of failures. These findings should not be generalized to all surgically treated eyes or to longer-term visual recovery. Larger studies with standardized imaging, reproducibility assessment and uniform longitudinal follow-up are required to determine whether selected OCT features provide incremental information beyond baseline VA and hole size.

## Figures and Tables

**Table 1:** Baseline clinical and OCT characteristics of the study cohort.

Characteristics	Overall (n = 127)
Number of patients	124
Bilateral inclusion, n (%)	3 (2.4%)
Patient-level characteristics	
Age at diagnosis, years ± SD	69.6 ± 7.6
Sex	
Male, n (%)	47 (37.9%)
Female, n (%)	77 (62.1%)
Eye-level characteristics	
Lens status	
Phakic, n (%)	91 (71.7%)
Pseudophakic, n (%)	36 (28.3%)
Symptom duration (days), median (IQR) Asymptomatic, n (%)	60 (30.0–120.0) 5 (3.9%)
Preoperative VA (logMAR) ± SD	0.79 ± 0.40
Preoperative OCT biomarkers	
Vitreomacular traction, n (%)	43 (33.9%)
Microcysts, n (%)	112 (88.2%)
Bumpy borders, n (%)	72 (56.7%)
Supra-RPE granular deposits, n (%)	60 (47.2%)
Epiretinal proliferation, n (%)	25 (19.7%)
Minimum linear diameter, µm ± SD	305.1 ± 175.8
Basal diameter, µm ± SD	783.3 ± 338.7
Foveal actual defect, µm ± SD	329.8 ± 191.9
ELM disruption, µm ± SD	402.9 ± 198.8
EZ disruption, µm ± SD	910.1 ± 440.4
CHW, µm ± SD	416.2 ± 198.4
IVTS classification	
Small (≤250 µm), n (%)	54 (42.5%)
Medium (>250 to ≤400 µm), n (%)	39 (30.7%)
Large (>400 µm), n (%)	34 (26.8%)

Data are presented as n (%), mean ± SD, or median (IQR). Symptom duration was available for 61 symptomatic eyes. Five eyes were asymptomatic, and symptom-duration information was unavailable for 61 eyes. Abbreviations: SD = standard deviation; VA = visual acuity; logMAR = logarithm of the minimum angle of resolution; OCT = optical coherence tomography; RPE = retinal pigment epithelium; ELM = external limiting membrane; EZ = ellipsoid zone; CHW = choroidal hypertransmission width; IVTS = international vitreomacular traction study.

**Table 2:** Adjusted Firth logistic regression models for primary failure.

Model/Predictor	OR	95% CI	*p* Value	Holm-Adjusted *p*
Core model				
MLD (per 100 µm)	2.09	1.46 to 3.26	<0.001	-
Core-plus-one-biomarker models				
Vitreomacular traction present	1.93	0.46 to 8.04	0.358	1.000
Microcysts present	0.09	0.01 to 0.71	0.024	0.239
Bumpy borders present	9.51	1.05 to 1255.25	0.043	0.390
Supra-RPE granular deposits present	0.60	0.14 to 2.41	0.469	1.000
Epiretinal proliferation present	0.58	0.05 to 3.88	0.571	1.000
Basal diameter (per 100 µm)	1.12	0.85 to 1.47	0.414	1.000
Foveal actual defect (per 100 µm)	1.09	0.49 to 2.06	0.801	1.000
ELM disruption (per 100 µm)	0.94	0.36 to 1.84	0.879	1.000
EZ disruption (per 100 µm)	0.97	0.77 to 1.21	0.803	1.000
CHW (per 100 µm)	2.02	1.02 to 4.31	0.045	0.390

Odds ratios with 95% CIs are from Firth penalized logistic regression with profile-likelihood CIs. *p* values are from penalized likelihood-ratio tests. Each biomarker was entered separately into a core model containing MLD. Holm-adjusted *p* values are shown for the exploratory biomarker analyses only. Bumpy borders were present in 11 of 11 eyes with primary failure and 61 of 116 eyes with primary closure. Estimates for bumpy borders should be interpreted cautiously because of the sparse outcome distribution and resulting imprecision. Abbreviations: MLD = minimum linear diameter; OR = odds ratio; CI = confidence interval; RPE = retinal pigment epithelium; ELM = external limiting membrane; EZ = ellipsoid zone; CHW = choroidal hypertransmission width.

**Table 3:** Postoperative outcomes by follow-up interval in all eyes.

Characteristic	21–60 Days (n = 107)	61–180 Days (n = 9)	181–365 Days (n = 9)	>365 Days (n = 22)
Follow-up days, mean (range)	28.0 (21.0–59.0)	97.1 (64.0–161.0)	259.1 (185.0–357.0)	991.4 (395.0–2080.0)
Postoperative VA (logMAR) ± SD	0.47 ± 0.34	0.31 ± 0.26	0.18 ± 0.12	0.35 ± 0.58
VA change from baseline (logMAR) ± SD	−0.34 ± 0.38	−0.49 ± 0.51	−0.54 ± 0.27	−0.42 ± 0.42
ELM disruption, µm ± SD	152.0 ± 246.6	130.0 ± 233.8	144.1 ± 287.5	125.0 ± 261.6
ELM change from baseline, µm ± SD	−265.5 ± 199.4	−192.0 ± 226.8	−262.9 ± 118.4	−226.2 ± 254.7
EZ disruption, µm ± SD	475.3 ± 471.0	303.8 ± 432.9	243.3 ± 374.2	217.9 ± 384.2
EZ change from baseline, µm ± SD	−455.7 ± 383.5	−398.3 ± 488.9	−746.1 ± 415.4	−678.3 ± 388.4
CHW, µm ± SD	128.8 ± 203.6	33.0 ± 99.0	74.8 ± 126.0	130.6 ± 296.1
CHW change from baseline, µm ± SD	−298.4 ± 201.1	−367.8 ± 199.8	−283.6 ± 120.8	−235.8 ± 288.5

Data are presented as mean ± SD unless otherwise indicated. Each eye could contribute to more than one follow-up interval if data were available at multiple visits; within each interval, only 1 visit per eye was retained. The 107 eyes assessed between postoperative days 21 and 60 comprised 99 eyes with successful primary closure and 8 eyes with primary failure. The conditional functional regression analysis included only the 99 successfully closed eyes. Abbreviations: VA = visual acuity; logMAR = logarithm of the minimum angle of resolution; SD = standard deviation; ELM = external limiting membrane; EZ = ellipsoid zone; CHW = choroidal hypertransmission width.

**Table 4:** Univariable and adjusted associations with early postoperative visual acuity among eyes with successful primary closure.

Model/Predictor	Univariable β (95% CI)	*p* Value	Holm-Adjusted *p*	Adjusted β (95% CI)	*p* Value	Holm-Adjusted *p*
Core model						
Baseline VA (per 0.1 logMAR)	0.0349 (0.0214 to 0.0484)	<0.001	-	0.0214 (−0.0017 to 0.0446)	0.069	-
MLD (per 100 µm)	0.0788 (0.0285 to 0.1292)	0.002	-	0.0465 (−0.0313 to 0.1243)	0.238	-
Core-plus-one-biomarker models						
Vitreomacular traction present	−0.0678 (−0.1898 to 0.0543)	0.273	0.546	−0.0292 (−0.1370 to 0.0786)	0.592	1.000
Microcysts present	0.1578 (0.0539 to 0.2616)	0.003	0.023	0.0029 (−0.1039 to 0.1097)	0.958	1.000
Bumpy borders present	0.1797 (0.0665 to 0.2929)	0.002	0.018	0.1076 (0.0100 to 0.2053)	0.031	0.311
Supra-RPE granular deposits present	0.0290 (−0.0943 to 0.1523)	0.642	0.642	−0.0696 (−0.1669 to 0.0278)	0.159	1.000
Epiretinal proliferation present	−0.1481 (−0.2592 to −0.0371)	0.009	0.047	−0.1043 (−0.2131 to 0.0045)	0.060	0.540
Basal diameter (per 100 µm)	0.0404 (0.0095 to 0.0712)	0.011	0.047	0.0175 (−0.0189 to 0.0539)	0.343	1.000
Foveal actual defect (per 100 µm)	0.0738 (0.0302 to 0.1174)	0.001	0.011	0.0260 (−0.0288 to 0.0808)	0.348	1.000
ELM disruption (per 100 µm)	0.0659 (0.0247 to 0.1070)	0.002	0.018	−0.0081 (−0.0730 to 0.0569)	0.806	1.000
EZ disruption (per 100 µm)	0.0255 (0.0081 to 0.0429)	0.005	0.027	0.0075 (−0.0074 to 0.0224)	0.322	1.000
CHW (per 100 µm)	0.0653 (0.0127 to 0.1179)	0.015	0.047	−0.0154 (−0.1059 to 0.0751)	0.737	1.000

Adjusted biomarker estimates were obtained from separate models containing baseline VA, MLD and one biomarker. Adjusted estimates for baseline VA and MLD are from the prespecified core model. Positive coefficients indicate worse postoperative logMAR VA. Patient-level cluster-robust standard errors accounted for bilateral inclusion. Holm adjustment was applied separately to the family of biomarker analyses and was not applied to baseline VA or MLD. All models included 99 eyes from 97 patients. Abbreviations: CI = confidence interval; VA = visual acuity; logMAR = logarithm of the minimum angle of resolution; RPE = retinal pigment epithelium; ELM = external limiting membrane; EZ = ellipsoid zone; CHW = choroidal hypertransmission width; MLD = minimum linear diameter.

## Data Availability

The data presented in this study are available from the corresponding author upon reasonable request. The data are not publicly available because they contain potentially identifiable clinical information.

## Supplementary Materials

The following supporting information can be downloaded at: https://www.mdpi.com/article/10.3390/diagnostics16203319/s1, Table S1, selected previous studies evaluating OCT biomarkers as predictors of anatomical or visual outcomes after full-thickness macular hole surgery; Table S2, operational definitions and assessment of preoperative OCT biomarkers; Table S3, baseline clinical and OCT characteristics stratified by closure status; Table S4, baseline and perioperative characteristics of successfully closed eyes according to inclusion in the early postoperative visual-acuity analysis; Table S5, postoperative outcomes by follow-up interval in eyes with primary closure; Tables S6A–S6D, supplementary analyses of early postoperative visual acuity; Table S7, exploratory associations between symptom duration and baseline OCT biomarkers.

## Abbreviations

BD	Basal diameter
C3F8	Perfluoropropane
CHW	Choroidal hypertransmission width
CI	Confidence interval
ELM	External limiting membrane
ERP	Epiretinal proliferation
EZ	Ellipsoid zone
FAD	Foveal actual defect
FTMH	Full-thickness macular hole
ILM	Internal limiting membrane
IQR	Interquartile range
logMAR	Logarithm of the minimum angle of resolution
MLD	Minimum linear diameter
OCT	Optical coherence tomography
OR	Odds ratio
PPV	Pars plana vitrectomy
RPE	Retinal pigment epithelium
SD	Standard deviation
SF6	Sulfur hexafluoride
VA	Visual acuity
VMT	Vitreomacular traction
