# Supplementary material for: Baseline OCT Biomarkers and Early Outcomes After Idiopathic Full-Thickness Macular Hole Surgery: Associations with Primary Failure and Visual Acuity After Successful Closure
Source: Diagnostics (Basel). 2026 Oct 13;16(20):3319. doi: 10.3390/diagnostics16203319 (PMC13703351; doi:10.3390/diagnostics16203319)
Supplement: Supplementary file 1 [file diagnostics-16-03319-s001.zip › diagnostics-4603675-supplementary.pdf]

Supplementary Table S1. Selected previous studies evaluating OCT biomarkers beyond MLD as predictors of anatomical or visual outcomes after FTMH surgery

Study	n	OCT biomarker	Outcome / follow-up	Key finding
**Akai et al., 2025 [7]**	94	VMT	VA, closure, and outer retinal recovery; 12 months	No significant difference in postoperative VA was found between eyes with and without focal VMT, and all eyes achieved primary closure. Eyes without focal VMT had poorer ELM recovery at 1 month, despite similarly favorable final outcomes.
**Joo et al., 2022 [6]**	35	Intraretinal cyst height	Closure type and photoreceptor integrity; 6 months	Greater intraretinal cyst height was associated with type 1 closure ($P = 0.011$) and intact photoreceptors after closure ($P = 0.046$). Intraretinal cyst height showed an AUC of 0.821 for predicting closure type.
**Govetto et al., 2023 [8]**	149	Supra-RPE granular deposits; bumpy borders	VA and ELM/EZ restoration; 12 months	Bumpy borders remained associated with worse postoperative VA after adjustment ($P < 0.001$) and with poorer outer retinal restoration. Supra-RPE granular deposits were associated with worse VA only in univariable analysis and were no longer significant after adjustment ($P = 0.320$).
**Kim et al., 2019 [9]**	113	Epiretinal proliferation	VA improvement and repeat surgery for failed closure; 12 months	Epiretinal proliferation remained associated with less VA improvement after adjustment ($\beta = -0.128$; $P = 0.046$) and markedly increased the likelihood of repeat surgery for failed closure (OR = 10.57; 95% CI, 1.49–74.92; $P = 0.009$).
**Houly et al., 2017 [10]**	52	Preoperative ELM, EZ and IZ defect lengths	VA; 3 and 6 months	Preoperative ELM, EZ, and IZ defect lengths were associated with postoperative VA. ELM defect length showed the strongest association with 6-month VA in multivariable analysis ($\beta = 0.643$; $P = 0.012$), while ELM integrity at 3 months was also associated with 6-month VA ($\beta = 0.427$; $P = 0.004$).
**Mehta et al., 2021 [13]**	44	CHW, EZ disruption	VA; mean 17 months	CHW correlated with postoperative VA ($r = 0.503$; $P = 0.0005$), slightly more strongly than MLD ($r = 0.491$; $P = 0.0007$). EZ disruption width was not associated with postoperative VA, whereas en face EZ loss area showed a weaker correlation ($r = 0.380$; $P = 0.013$).
**Alkabes et al., 2024 [21]**	36	CHW, ΔCHW	Postoperative VA; median 9 months	CHW was associated with postoperative VA (0.008 logMAR per 10 μm; $P < 0.001$). When modeled with MLD, ΔCHW remained independently associated with postoperative VA ($\beta = 0.333$; $P = 0.025$).
**Mase et al., 2024 [20]**	43	ONL and OPL defect-length metrics	VA classification at 6 months	A model combining preoperative VA with OCT-derived morphology achieved an AUC of 0.84. The OPL–ONL defect-length difference contributed to prediction (coefficient −0.212), although adding OCT features only slightly improved performance over clinical information alone (AUC 0.82).
**Ruban et al., 2025 [16]**	57	FAD	Primary closure by day 14 and VA at 6 months	FAD <400 μm was associated with a higher probability of closure by day 14 (RR = 1.30; 95% CI, 1.00–1.60; $P = 0.02$). FAD also correlated with 6-month VA ($\rho = 0.37$; $P = 0.002$), and a model including baseline VA, FAD, and basal diameter explained 75% of the variance in postoperative VA ($R^2 = 0.75$).

Effect estimates are reported according to the statistical approach used in the original studies. Odds ratios were not available for all studies because several evaluated postoperative visual acuity as a continuous outcome using correlation or linear regression.

Abbreviations: AUC = area under the curve; CHW = choroidal hypertransmission width; ELM = external limiting membrane; EZ = ellipsoid zone; FAD = foveal actual defect; IZ = interdigitation zone; MLD = minimum linear diameter; ONL = outer nuclear layer; OPL = outer plexiform layer; OR = odds ratio; RPE = retinal pigment epithelium; RR = relative risk; VA = visual acuity; VMT = vitreomacular traction

Supplementary Table S2. Operational definitions and assessment of preoperative OCT biomarkers

OCT feature	Type	Operational definition	Assessment method
**Vitreomacular traction**	Binary	Persistent focal attachment of the posterior hyaloid to the macula with associated distortion, elevation, or deformation of the foveal tissue.	Present or absent across all available radial B-scans.
**Microcysts**	Binary	One or more well-demarcated hyporeflective intraretinal cystic spaces within the elevated retinal tissue adjacent to the macular hole.	Present or absent across all available radial B-scans.
**Bumpy borders**	Binary	Irregular or undulating macular-hole margins with visible projections, lobulations, or indentations.	Present or absent on the B-scan or scans showing the clearest hole-margin morphology.
**Supra-RPE granular deposits**	Binary	Discrete hyperreflective granular foci located immediately above the retinal pigment epithelium at the base of the macular hole.	Present or absent across the central and adjacent B-scans.
**Epiretinal proliferation**	Binary	Homogeneous material of intermediate reflectivity on the inner retinal surface, contiguous with the macular-hole margin and distinguishable from a conventional hyperreflective tractional epiretinal membrane.	Present or absent across all available radial B-scans.
**Minimum linear diameter**	Continuous, μm	The narrowest full-thickness distance between the opposing retinal edges of the macular hole, measured parallel to the retinal pigment epithelium.	Measured manually using HEYEX calipers on the B-scan showing the narrowest hole aperture.
**Basal diameter**	Continuous, μm	The linear distance between the nasal and temporal points at which retinal detachment from the retinal pigment epithelium began at the base of the macular hole.	Measured manually using HEYEX calipers on the selected foveal B-scan.
**Foveal actual defect**	Continuous, μm	Calculated as basal diameter minus the sum of the detached nasal and temporal retinal lengths: FAD = BD − (nasal detached retinal length + temporal detached retinal length). Each detached retinal length extended from the perpendicular projection at the beginning of retinal detachment to the end of the detached retinal tissue along the external limiting membrane contour.	Measured manually on the foveal horizontal B-scan using HEYEX calipers.
**External limiting membrane disruption**	Continuous, μm	The horizontal distance between the nearest clearly identifiable ends of the external limiting membrane on either side of the macular hole.	Measured parallel to the retinal pigment epithelium using HEYEX calipers on the selected foveal B-scan.
**Ellipsoid zone disruption**	Continuous, μm	The horizontal distance between the nearest clearly identifiable ends of the ellipsoid zone on either side of the macular hole.	Measured parallel to the retinal pigment epithelium using HEYEX calipers on the selected foveal B-scan.
**Choroidal hypertransmission width**	Continuous, μm	The horizontal width of increased OCT signal transmission into the choroid beneath the macular hole, bounded by the transition between normal and visibly increased choroidal transmission.	Measured manually using HEYEX calipers on the B-scan showing the clearest extent of hypertransmission.

Qualitative features were assessed across the available preoperative B-scans. Quantitative measurements were obtained manually using the device caliper tool according to the anatomical landmarks defined above. FAD was calculated as described by Ruban et al. (reference 16 in the main manuscript).
Abbreviations: BD = basal diameter; ELM = external limiting membrane; EZ = ellipsoid zone; FAD = foveal actual defect; OCT = optical coherence tomography; RPE = retinal pigment epithelium; VMT = vitreomacular traction.

Supplementary Table S3. Baseline clinical and OCT characteristics stratified by closure status

Characteristics	Primary closure (n = 116)	Primary failure (n = 11)
Number of patients	113	11
**Patient-level characteristics**		
Age at diagnosis, years ± SD	69.1 ± 7.5	76.0 ± 6.3
Sex		
Male, n (%)	43 (38.1%)	4 (36.4%)
Female, n (%)	70 (61.9%)	7 (63.6%)
**Eye-level characteristics**		
Lens status		
Phakic, n (%)	84 (72.4%)	7 (63.6%)
Pseudophakic, n (%)	32 (27.6%)	4 (36.4%)
Symptom duration (days), median (IQR)	60 (30.0–120.0)	150.0 (45.0–293.8)
Asymptomatic, n (%)	5 (4.3%)	0 (0.0%)
Preoperative VA (logMAR) ± SD	0.76 ± 0.37	1.09 ± 0.55
**Preoperative OCT biomarkers**		
Vitreomacular traction, n (%)	39 (33.6%)	4 (36.4%)
Microcysts, n (%)	103 (88.8%)	9 (81.8%)
Bumpy borders, n (%)	61 (52.6%)	11 (100.0%)
Supra-RPE granular deposits, n (%)	54 (46.6%)	6 (54.5%)
Epiretinal proliferation, n (%)	24 (20.7%)	1 (9.1%)
Minimum linear diameter, μm ± SD	283.8 ± 165.8	529.1 ± 113.9
Basal diameter, μm ± SD	746.8 ± 318.0	1168.1 ± 322.6
Foveal actual defect, μm ± SD	308.2 ± 177.3	558.3 ± 198.8
ELM disruption, μm ± SD	381.4 ± 187.0	630.1 ± 183.0
EZ disruption, μm ± SD	882.4 ± 442.2	1202.1 ± 304.7
CHW, μm ± SD	389.8 ± 179.8	693.7 ± 177.0
**IVTS classification**		
Small (≤250 μm), n (%)	54 (46.6%)	0 (0.0%)
Medium (>250 to ≤400 μm), n (%)	37 (31.9%)	2 (18.2%)
Large (>400 μm), n (%)	25 (21.6%)	9 (81.8%)

Data are presented as n (%), mean ± SD, or median (IQR). Symptom duration was available for 53 symptomatic eyes with primary closure and 8 symptomatic eyes with primary failure. Five eyes were asymptomatic, and symptom-duration information was unavailable for 58 eyes with primary closure and 3 eyes with primary failure.

Abbreviations: SD = standard deviation; VA = visual acuity; logMAR = logarithm of the minimum angle of resolution; OCT = optical coherence tomography; RPE = retinal pigment epithelium; ELM = external limiting membrane; EZ = ellipsoid zone; CHW = choroidal hypertransmission width; IVTS = international vitreomacular traction study.

Supplementary Table S4. Baseline and perioperative characteristics of successfully closed eyes according to inclusion in the early postoperative visual-acuity analysis

Characteristic	Included (n = 99)	Excluded (n = 17)	Absolute SMD
Age at diagnosis, years	69.5 ± 7.5	66.4 ± 6.4	0.45
Female sex, n (%)	58 (58.6%)	13 (76.5%)	0.39
Phakic at baseline, n (%)	70 (70.7%)	14 (82.4%)	0.28
Preoperative VA, logMAR	0.79 ± 0.38	0.61 ± 0.28	0.52
Vitreomacular traction present, n (%)	29 (29.3%)	10 (58.8%)	0.62
Microcysts present, n (%)	88 (88.9%)	15 (88.2%)	0.02
Bumpy borders present, n (%)	54 (54.5%)	7 (41.2%)	0.27
Supra-RPE granular deposits present, n (%)	49 (49.5%)	5 (29.4%)	0.42
Epiretinal proliferation present, n (%)	20 (20.2%)	4 (23.5%)	0.08
Minimum linear diameter, µm	295.2 ± 166.8	217.9 ± 147.4	0.49
Basal diameter, µm	766.0 ± 313.8	634.8 ± 328.5	0.41
Foveal actual defect, µm	324.0 ± 177.5	215.8 ± 149.7	0.66
ELM disruption, µm	396.5 ± 189.5	293.6 ± 147.7	0.61
EZ disruption, µm	906.2 ± 441.9	744.2 ± 430.9	0.37
Choroidal hypertransmission width, µm	403.9 ± 182.7	307.9 ± 140.0	0.59
SF6 tamponade, n (%)	91 (91.9%)	17 (100.0%)	0.42
Face-down positioning, days	4.6 ± 2.1	5.4 ± 2.9	0.32

Data are presented as n (%) or mean ± SD. Included eyes had successful primary closure and an eligible visual-acuity assessment between postoperative days 21 and 60. When more than one eligible visit was available, the visit closest to postoperative day 30 was selected. Absolute standardized mean differences are presented descriptively, with larger values indicating greater observed imbalance between groups. No formal hypothesis testing was performed because this comparison was intended to assess potential selection into the functional analysis. All phakic eyes underwent combined phacoemulsification at the time of macular hole surgery. No data were missing for the variables shown.

Abbreviations: CHW = choroidal hypertransmission width; ELM = external limiting membrane; EZ = ellipsoid zone; FAD = foveal actual defect; logMAR = logarithm of the minimum angle of resolution; MLD = minimum linear diameter; RPE = retinal pigment epithelium; SD = standard deviation; SF6 = sulfur hexafluoride; SMD = standardized mean difference; VA = visual acuity; VMT = vitreomacular traction.

Supplementary Table S5. Postoperative outcomes by follow-up interval in eyes with primary closure

Characteristic	21–60 days (n = 99)	61–180 days (n = 7)	181–365 days (n = 9)	>365 days (n = 19)
Follow-up days, mean (range)	27.4 (21.0–59.0)	105.0 (64.0–161.0)	259.1 (185.0–357.0)	955.3 (395.0–1818.0)
Postoperative VA (logMAR) ± SD	0.43 ± 0.31	0.20 ± 0.15	0.18 ± 0.12	0.17 ± 0.14
VA change from baseline (logMAR) ± SD	-0.36 ± 0.37	-0.48 ± 0.32	-0.54 ± 0.27	-0.52 ± 0.24
ELM disruption, µm ± SD	106.3 ± 180.2	14.3 ± 26.3	144.1 ± 287.5	60.6 ± 178.5
ELM change from baseline, µm ± SD	-290.2 ± 173.0	-277.4 ± 167.5	-262.9 ± 118.4	-261.2 ± 237.2
EZ disruption, µm ± SD	401.2 ± 381.2	89.0 ± 87.8	243.3 ± 374.2	118.7 ± 251.5
EZ change from baseline, µm ± SD	-505.0 ± 316.0	-562.1 ± 383.4	-746.1 ± 415.4	-714.8 ± 389.6
CHW, µm ± SD	94.3 ± 156.0	0.0 ± 0.0	74.8 ± 126.0	50.4 ± 201.9
CHW change from baseline, µm ± SD	-309.6 ± 198.1	-334.7 ± 217.9	-283.6 ± 120.8	-288.3 ± 260.1

Data are presented as mean ± SD unless otherwise indicated. Each eye could contribute to more than one follow-up interval if data were available at multiple visits; within each interval, only 1 visit per eye was retained.

Abbreviations: VA = visual acuity; logMAR = logarithm of the minimum angle of resolution; SD = standard deviation; ELM = external limiting membrane; EZ = ellipsoid zone; CHW = choroidal hypertransmission width.

Supplementary Tables S6A–S6D. Supplementary analyses of early postoperative visual acuity

Table S6A. Fully adjusted sensitivity models

Biomarker	β (95% CI)	P value	Holm-adjusted P
Vitreomacular traction present	-0.0245 (-0.1409 to 0.0919)	0.677	1.000
Microcysts present	0.0160 (-0.1077 to 0.1397)	0.798	1.000
Bumpy borders present	0.1167 (0.0164 to 0.2169)	0.023	0.230
Supra-RPE granular deposits present	-0.0905 (-0.1927 to 0.0118)	0.082	0.742
Epiretinal proliferation present	-0.0941 (-0.2078 to 0.0196)	0.104	0.831
Basal diameter (per 100 µm)	0.0243 (-0.0158 to 0.0643)	0.232	1.000
Foveal actual defect (per 100 µm)	0.0316 (-0.0309 to 0.0942)	0.318	1.000
ELM disruption (per 100 µm)	-0.0070 (-0.0755 to 0.0615)	0.840	1.000
EZ disruption (per 100 µm)	0.0101 (-0.0049 to 0.0251)	0.186	1.000
Choroidal hypertransmission width (per 100 µm)	-0.0128 (-0.1119 to 0.0863)	0.798	1.000

Each model included baseline VA, MLD, age, baseline phakic status, tamponade type, exact postoperative assessment day, and one OCT biomarker. Positive coefficients indicate worse postoperative logMAR VA. Patient-level cluster-robust standard errors were used. All models included 99 eyes from 97 patients.

Table S6B. Incremental model performance after adding individual OCT biomarkers

Biomarker	Extended adjusted R^2	Δ adjusted R^2	ΔAIC	ΔRMSE
Vitreomacular traction present	0.201	-0.006	1.76	-0.0003
Microcysts present	0.199	-0.008	2.00	0.0000
Bumpy borders present	0.228	0.021	-1.65	-0.0049
Supra-RPE granular deposits present	0.211	0.004	0.51	-0.0020
Epiretinal proliferation present	0.218	0.011	-0.38	-0.0032
Basal diameter (per 100 µm)	0.212	0.005	0.37	-0.0022
Foveal actual defect (per 100 µm)	0.203	-0.004	1.48	-0.0007
ELM disruption (per 100 µm)	0.199	-0.008	1.96	-0.0001
EZ disruption (per 100 µm)	0.206	-0.001	1.14	-0.0012
Choroidal hypertransmission width (per 100 µm)	0.200	-0.007	1.82	-0.0002

The core model included baseline VA and MLD and had an adjusted R^2 of 0.207, AIC of 27.10, and RMSE of 0.269. Each extended model added one biomarker to the core model. All comparisons used the same 99 eyes. Negative ΔAIC and ΔRMSE indicate improved model fit.

Table S6C. Pearson correlations among baseline visual acuity and continuous OCT measures

Variable	Baseline VA	MLD	BD	FAD	ELM	EZ	CHW
Baseline VA	1.00	0.66	0.55	0.64	0.67	0.56	0.65
MLD	0.66	1.00	0.78	0.90	0.93	0.62	0.91
BD	0.55	0.78	1.00	0.72	0.75	0.73	0.82
FAD	0.64	0.90	0.72	1.00	0.94	0.56	0.87
ELM	0.67	0.93	0.75	0.94	1.00	0.66	0.92
EZ	0.56	0.62	0.73	0.56	0.66	1.00	0.67
CHW	0.65	0.91	0.82	0.87	0.92	0.67	1.00

All correlations were based on 99 eyes. Spearman correlations showed a similar pattern. Baseline VA and MLD had Pearson $r = 0.66$ and Spearman $\rho = 0.61$ (both $P < 0.001$).

Table S6D. Variance inflation factors for the core and core-plus-one-biomarker models

Model	Baseline VA VIF	MLD VIF	Added biomarker VIF
Core	1.77	1.77	—
Core + Vitreomacular traction present	1.77	1.79	1.02
Core + Microcysts present	1.78	1.87	1.14
Core + Bumpy borders present	1.78	1.84	1.09
Core + Supra-RPE granular deposits present	1.79	1.83	1.11
Core + Epiretinal proliferation present	1.79	1.77	1.02
Core + Basal diameter (per 100 μm)	1.78	3.15	2.53
Core + Foveal actual defect (per 100 μm)	1.81	5.73	5.51
Core + ELM disruption (per 100 μm)	1.84	7.57	7.72
Core + EZ disruption (per 100 μm)	1.89	2.13	1.74
Core + Choroidal hypertransmission width (per 100 μm)	1.83	6.02	5.94

Variance inflation factors were calculated using the same 99 eyes included in each functional model. Higher VIFs in models containing FAD, ELM disruption, or CHW reflect substantial overlap with MLD.

Abbreviations: AIC = Akaike information criterion; BD = basal diameter; CHW = choroidal hypertransmission width; CI = confidence interval; ELM = external limiting membrane; EZ = ellipsoid zone; FAD = foveal actual defect; MLD = minimum linear diameter; OCT = optical coherence tomography; RMSE = root mean square error; VA = visual acuity; VIF = variance inflation factor.

Supplementary Table S7. Exploratory associations between symptom duration and baseline OCT biomarkers

Table S7A. Associations between symptom duration and quantitative OCT biomarkers

Biomarker	N	Spearman ρ	P value	Holm-adjusted P
Minimum linear diameter	61	0.321	0.012	0.066
Basal diameter	61	0.506	<0.001	<0.001
Foveal actual defect	61	0.387	0.002	0.018
ELM disruption	61	0.399	0.001	0.014
EZ disruption	61	0.350	0.006	0.046
Choroidal hypertransmission width	61	0.325	0.011	0.066

Table S7B. Associations between symptom duration and qualitative OCT biomarkers

Biomarker	Features absent, n	Features present, n	Symptom duration if absent, median (IQR)	Symptom duration if present, median (IQR)	P value	Holm-adjusted P
Vitreomacular traction	40	21	90 (40–180)	50 (30–90)	0.009	0.066
Microcysts	5	56	30 (6–30)	67.5 (38.8–127.5)	0.018	0.071
Bumpy borders	25	36	60 (30–120)	62.5 (43.8–120)	0.571	1.000
Supra-RPE granular deposits	30	31	75 (38.8–120)	60 (30–120)	0.638	1.000
Epiretinal proliferation	50	11	60 (30–120)	120 (52.5–155)	0.240	0.720

Analyses were restricted to the 61 symptomatic eyes with documented symptom duration. Associations with continuous OCT measures were assessed using Spearman rank correlation. Symptom duration was compared between eyes with and without qualitative OCT features using the Mann–Whitney U test. Holm adjustment was applied across the 11 exploratory biomarker analyses.

Abbreviations: IQR = interquartile range; OCT = optical coherence tomography; ELM = external limiting membrane; EZ = ellipsoid zone.
